# HAF mediates the evasive resistance of anti-angiogenesis TKI through disrupting HIF-1α and HIF-2α balance in renal cell carcinoma

**DOI:** 10.18632/oncotarget.17923

**Published:** 2017-05-17

**Authors:** Xiang-Me Lai, Shu-Yu Liu, Yi-Ta Tsai, Guang-Huan Sun, Sun-Yran Chang, Shih-Ming Huang, Tai-Lung Cha

**Affiliations:** ^1^ Graduate Institute of Life Sciences, National Defense Medical Center, Taipei, Taiwan, R.O.C; ^2^ Division of Urology, Department of Surgery, Tri-Service General Hospital, National Defense Medical Center, Taipei, Taiwan, R.O.C; ^3^ Graduate Institute of Medical Sciences, National Defense Medical Center, Taipei, Taiwan, R.O.C; ^4^ Buddhist Tzu Chi General Hospital, Taipei, Taiwan, R.O.C; ^5^ Department of Biochemistry, National Defense Medical Center, Taipei, Taiwan, R.O.C

**Keywords:** tyrosine kinase inhibitor, hypoxia-inducible factor, hypoxia associated factor, angiogenesis, renal cell carcinoma

## Abstract

Anti-angiogenesis has emerged as a standard of care for metastatic renal cell carcinoma. However, long-lasting efficacy is seldom reached, and evasive resistance eventually occurs under anti-angiogenic tyrosine kinase inhibitor (TKI) therapy. To establish new therapeutic strategies, investigating the molecular mechanism of resistance is critically important. In our study, human umbilical vascular endothelial cells (HUVECs) were incubated with TKI treatment in conditioned medium derived from renal cancer cells (RCCs) to demonstrate cell viability. Quantitative real time PCR or Western blotting analysis detected the fluctuation of transcriptional factors HIF-1α and HIF-2α in RCCs under TKI treatment. We demonstrated the alteration of a specific cytokine produced from RCCs under normoxia or hypoxia incubation by utilizing a cytokine RT-PCR primer array. We found that the anti-angiogenic TKI sunitinib disrupted the balance between HIF-1α and HIF-2α in RCCs and led to a protective effect on HUVECs against sunitinib treatment when cultured with conditioned medium. Mechanistically, RCCs treated with sunitinib resulted in down-regulation of HIF-1α, but not HIF-2α, through reduction of both mRNA and protein levels. The down-regulation of HIF-1α by sunitinib occurred via hypoxia associated factor (HAF), which also enhanced HIF-2α transactivation activity to increase the production of pro-angiogenic factors and cytokines and promote HUVEC proliferation. This phenomenon was observed in ACHN and A498 cells, which express both HIF-1α and HIF-2α, but was not observed in 786-O cells, which express only HIF-2α. Our results illustrated that targeting both angiogenesis and hypoxia pathways might provide a resolution to dealing with the devastating effects of anti-angiogenesis resistance.

## INTRODUCTION

Anti-angiogenesis has emerged as the standard care for metastatic renal cell carcinoma [1]. Anti-angiogenic multiple tyrosine kinase inhibitors (TKIs) such as sunitinib, sorafenib, pazopanib and axitinib have been demonstrated to have clinical benefits in the treatment of metastatic renal cell carcinoma in both first-line and second-line settings [2]. These TKIs have diverse molecular profiles and different affinities for vascular endothelial growth factor (VEGF) and platelet derived growth factor (PDGF) receptors to inhibit tumor angiogenesis. Common emerging problems include the lack of achieving complete and durable responses subsequent to the eventual development of evasive-resistance in the clinics [3, 4].

Recently, there are at least four distinct adaptive mechanisms for evasive resistance to antiangiogenic TKI therapy: first, activation and/or upregulation of alternative pro-angiogenic signaling pathways within the tumor; second, recruitment of bone marrow-derived pro-angiogenic cells; third, increased pericyte coverage of the tumor vasculature; and fourth, activation and enhancement of invasion and metastasis to provide access to normal tissue vasculature without obligate neovascularization. There is increasing evidence that pre-clinical and clinical observations that have evasive-resistance to anti-angiogenic TKIs are attributed to diverse molecular events [5, 6]. Although different cell types in the tumor microenvironment have fundamental effects on promoting angiogenesis, tumor cells remain the major contributor to the development of anti-angiogenic TKIs evasive-resistance [7, 8]. However, the detailed molecular mechanisms for how RCCs acquire evasive-resistance to TKI therapy remain unknown.

Hypoxia-inducible factors (HIFs) are critical transcriptional factors that mediate the hypoxic response for angiogenesis. HIFs form heterodimers comprising one of two major oxygen-labile a subunits (HIF-1α and HIF-2α) and a stable HIF-1β subunit, and turn on pro-angiogenic target genes upon hypoxia [9]. Although elevated levels of tumor HIF-1α and HIF-2α have been associated with poor patient survival in multiple tumor types [10–13], HIF-1α and HIF-2α have distinct functions regarding inhibition or promotion of cancer growth. RCCs expressing HIF-2α have increased proliferation through promotion of c-Myc transactivation activity; while RCCs expressing HIF-1α have inhibited cell-cycle progression through repression of c-Myc [14]. HIF-1α can function as a tumor suppressor by up-regulating genes such as *Ndrg1* and *Selenbp1*, whose loss is associated with poor prognosis in human epithelial malignancies [15]. Through comprehensive genome studies, arm level losses on chromosome 14q, associated with loss of HIF-1α, have been shown to drive more aggressive disease [16, 17]. Therefore, disruption of the balance between HIF-1α and HIF-2α by extracellular assaults may lead to HIF-2α-mediated oncogenic activation of target genes, contributing to cancer cell survival in stringent tumor microenvironments [18, 19].

Hypoxia associated factor (HAF) is a specific E3 ubiquitin ligase for HIF-1α protein degradation in an oxygen-independent manner [20]. HAF has been shown to be overexpressed in a variety of cancer types [21–24]. It has been shown that HAF switches the hypoxic response of the cancer cell from HIF-1α-dependent to HIF-2α-dependent transcription and activates genes involved in invasion such as *MMP9*, *PAI-1*, and the stem cell factor *OCT3/4* [23, 24]. HAF induces ubiquitination and proteasome degradation of HIF-1α protein, and subsequently binds to HIF-2α protein, which turns on its downstream target genes in long-term hypoxia [22]. The HAF-mediated switch to HIF-2α-dependent gene expression promotes the enrichment of the cancer stem cell population, resulting in more aggressive tumors *in vivo* [23]. By disrupting the balance between HIF-1α and HIF-2α upon longer exposure of hypoxia, HAF leads to a highly aggressive cancer phenotype.

In the present study, we demonstrated that anti-angiogenic TKIs, such as sunitinib, disrupted the balance between HIF-1α and HIF-2α due to the depletion of HIF-1α through mRNA suppression and protein degradation by the E3 ubiquitin ligase HAF. HIF-1α and HIF-2α mediate distinct cellular responses depending on the variability in hypoxic intensity and duration [21, 25]. In addition to its involvement in the disruption of the balance between HIF-1α and HIF-2α, HAF may also be involved in the regulation of HIF-2α-dependent transactivation for the growth protective effect of RCCs after sunitinib treatment. The subtle change in the ratio of HIF-1α and HIF-2α in cells mediated by the dual functions of HAF in hypoxia might provide a new strategy to develop a combination therapy for RCC.

## RESULTS

### Renal cancer cell lines have different potentials to protect endothelial cells against sunitinib

**Figure 1:**
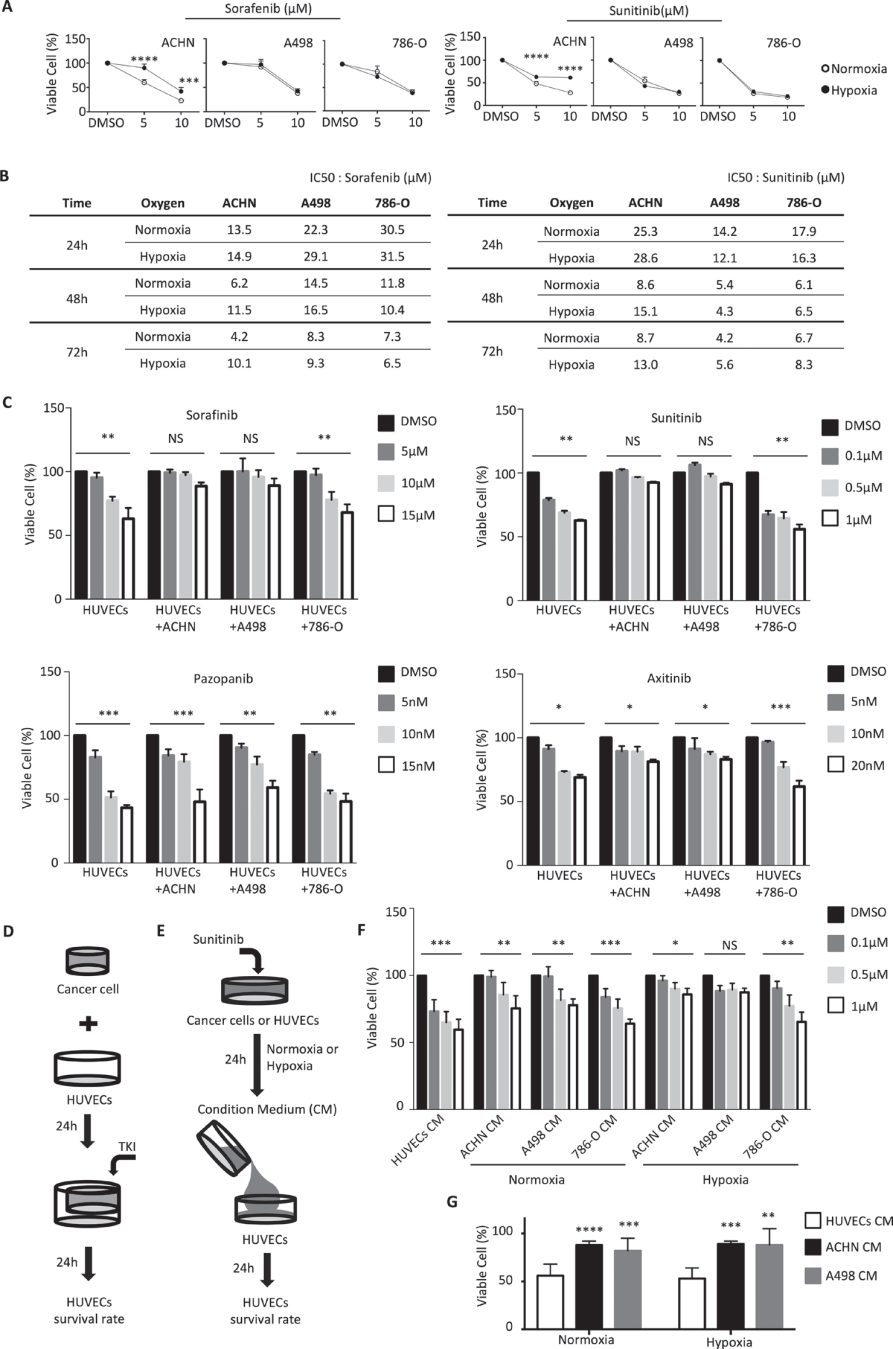
RCCs have different potentials to protect endothelial cells against sunitinib (**A**) ACHN, A498, and 786-O were incubated with sorafenib and sunitinib for 48 hours, after which cell viability was assessed by the SRB assay. The experiments were repeated three times. (**B**) Determination of IC50 values for sorafenib and sunitinib in ACHN, A498, and 786-O. (**C**) HUVECs viability in the co-culture system. Cell integrity in control cultures and the co-culture system was determined after 24 h of treatment with different dosages of sorafenib, sunitinib, axitinib or papzopanib. (**D**) Schematic representation of the co-culture experiments of HUVECs with indicated cell lines using cell culture inserts. (**E**) Schematic representation of the culture protocol for conditioned medium, RCCs treatment with different dosage sunitinib in normoxia or hypoxia for 24 h, and the culture medium (conditioned medium) treatment of HUVECs. (**F**) HUVECs were grown to confluence and were then cultured in conditioned medium (derived from indicated cell lines pretreated with different dosages of sunitinib under normoxia or hypoxia for 24 h) for 24 h. HUVECs alone were used as a control. (**G**) Comparison of viability of HUVECs incubated with conditioned medium derived from ACHN or A498 treated with 5 μM sunitinib. The result represents the mean ± S.D. (*P ≤ 0.05, **P ≤ 0.01, ***P ≤ 0.001, ****P ≤ 0.0001, NS: *P* > 0.05).

We examined the growth inhibition effects of varying doses of the anti-angiogenic TKIs sorafenib and sunitinib on the human RCC lines ACHN, A498, and 786-O. We observed that the growth rates of RCCs were inhibited by the TKIs in dosage- and time-dependent manners (Figure 1A and 1B). Under hypoxic growth conditions, the inhibition effects of the TKIs were significantly reduced for ACHN, as compared to normoxic conditions (Figure 1A and 1B). However, the inhibition effects of the TKIs on RCC lines A498 and 786-O were not significantly different between hypoxic and normoxic growth conditions (Figure 1A and 1B) The IC50 concentrations of the indicated TKIs were determined and used as the concentrations of choice for further studies (Figure 1B).

We next investigated the interaction between cancer cells and human umbilical vascular endothelial cells (HUVECs) under anti-angiogenic TKI treatment. We used a co-culture system to test the effects of TKI treatment on HUVEC cell growth in the presence or absence of RCCs (Figure 1D). Without co-cultured RCCs, the TKIs suppressed the growth rate of HUVECs in a dosage-dependent manner. Interestingly, the growth inhibition effects of sunitinib and of sorafenib, but not axitinib and pazopanib, on HUVECs were significantly compromised by co-culturing with RCC lines ACHN or A498 (Figure 1C). Co-culturing HUVECs with RCC lines ACHN or A498 provided a protective effect of about 30% against all tested concentrations of sunitinib and sorafenib (Figure 1C). Interestingly, co-culturing HUVECs with RCC line 786-O did not show any protective effect (Figure 1C).

We used the conditioned media taken from RCCs or HUVECs that were treated for 24h with TKIs for further studies (Figure 1E). Conditioned medium derived from A498 cells grown in hypoxic conditions provided a significant protective effect on HUVECs against up to 1 μM sunitinib (Figure 1F). Conditioned medium derived from ACHN and A498 was able to provide a protective effect to HUVECs against increased dosages of sunitinib (Figure 1G). These results suggested that different cancer cells have different capabilities to protect vascular endothelial cells against TKIs treatment.

### Down-regulation of HIF-1α by TKIs correlated with the protective effect of renal cancer cells that express both HIF-1α and HIF-2α

**Figure 2:**
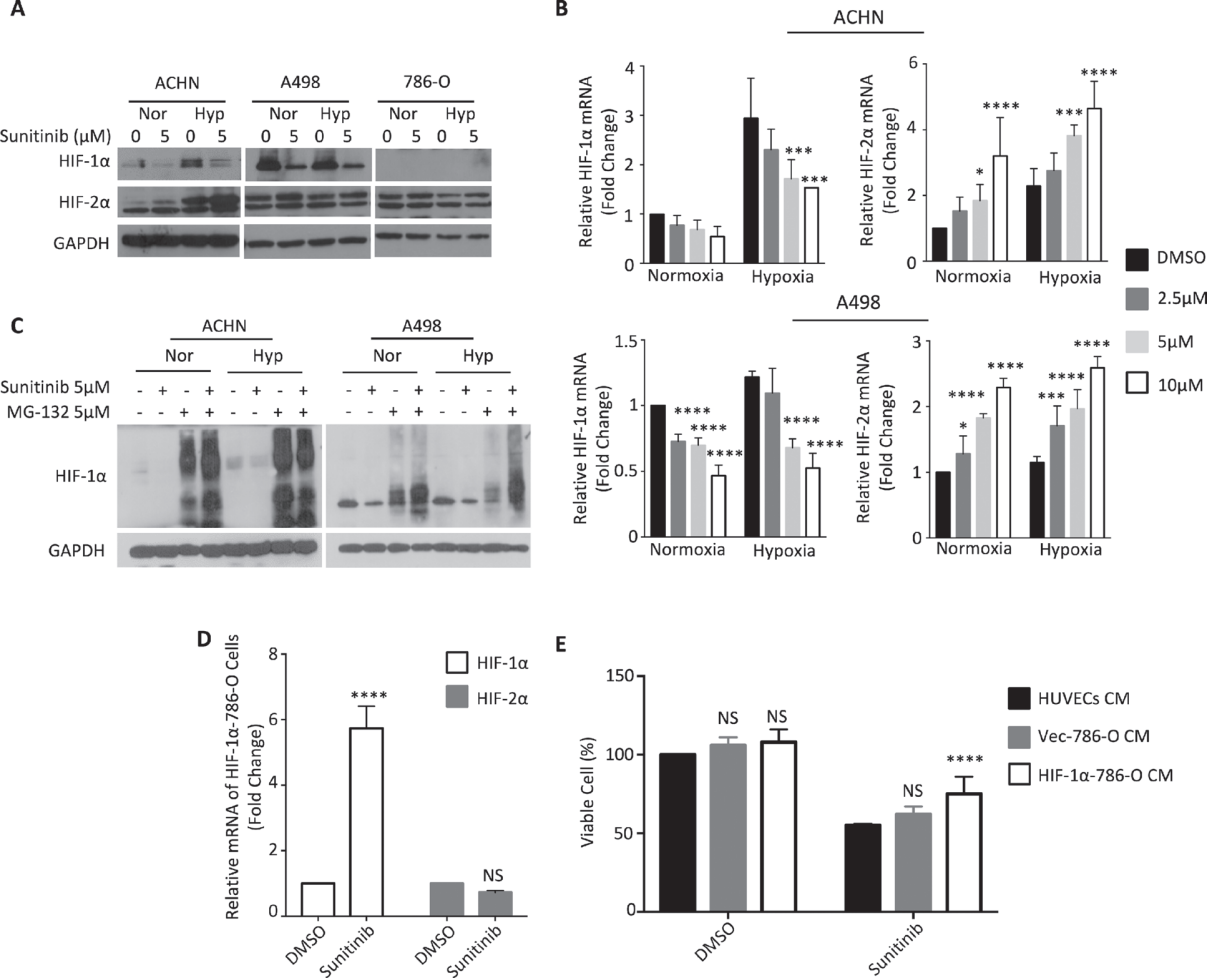
Sunitinib downregulates HIF-1α under normoxia or hypoxia ACHN, A498 and 786-O cells were incubated under normoxic (Nor) or hypoxic (Hyp) conditions in the presence of sunitinib for 24 h. (**A**) HIF-1α and HIF-2α proteins in total cell lysates were analyzed by Western blot. (**B**) The total mRNA was analyzed by quantitative real-time PCR. (**C**) ACHN and A498 cells were treated with 5 μM sunitinib and/or 5 μM MG-132 for 24 h and lysed for western blotting with HIF-1α antibody and GAPDH antibody (loading control). (**D**) 786-O cells were transfected with *HIF-1α* plasmid or control empty vector and treated with DMSO or 5 μM sunitinib for 24 hours. *HIF-1α* and *HIF-2α* mRNA levels were evaluated by using quantitative real-time *PCR*. (**E**) HUVECs were cultured with conditioned medium derived from 786-O cells transfected with *HIF-1α* plasmid or control empty vector and treated with DMSO or 5 μM sunitinib for 24 h. HUVECs viability was assessed by SRB assay. The results represent the means ± S.D. (*P ≤ 0.05, **P ≤ 0.01, ***P ≤ 0.001, ****P ≤ 0.0001, NS: *P* > 0.05).

From the above findings, the protective effect provided by RCCs against TKI treatment may be attributed to soluble factor(s) released from RCCs into the medium in hypoxic conditions. We chose HIFs to be our primary targets, because they have transactivation activities to regulate the expression of several cytokines [26, 27], and they are also responsible for hypoxic angiogenesis. We first examined the level of HIF protein using Western blot analysis, and observed that ACHN and A498 cells expressed both HIF-1α and HIF-2α, but that 786-O cells expressed only HIF-2α (Figure 2A).

Since sunitinib is one of the most common and widely used TKIs for the therapeutic treatment of renal cell carcinoma, we focused on sunitinib to validate the protective effect of RCC lines on HUVECs against TKI treatment. Sunitinib treatment reduced the level of HIF-1α protein, but did not affect the level of HIF-2α protein in ACHN and A498 cells (Figure 2A). We subsequently analyzed the mRNA levels of *HIF-1α* and *HIF-2α* in RCCs treated with sunitinib. Sunitinib treatment reduced *HIF-1α* mRNA levels and increased *HIF-2α* mRNA levels in a dosage-dependent manner in ACHN and A498 cells under normoxic and hypoxic conditions (Figure 2B). We treated ACHN and A498 cells with the proteasome inhibitor MG-132, and observed that MG-132 restored HIF-1α protein levels (Figure 2C). These results indicated that sunitinib treatment down-regulated HIF-1α protein levels by both reducing its mRNA expression and by degrading the HIF-1α protein itself.

We transfected *HIF-1α* into 786-O cells to further evaluate if the down-regulation of HIF-1α was responsible for the protective effect against TKI treatment. *HIF-1α* mRNA level was stably expressed under sunifinib treatment, and HIF-2α mRNA level was sustained (Figure 2D). Overexpression of *HIF-1α* in 786-O cells enhanced their ability to rescue the sunitinib-induced cell death in HUVECs via conditioned medium system (Figure 2E). We speculate that disruption of the balance between HIF-1α and HIF-2α through down-regulation of HIF-1α expression by sunitinib may lead to the protective effect.

### HIF-2α is responsible for the protective effect against sunitinib

**Figure 3:**
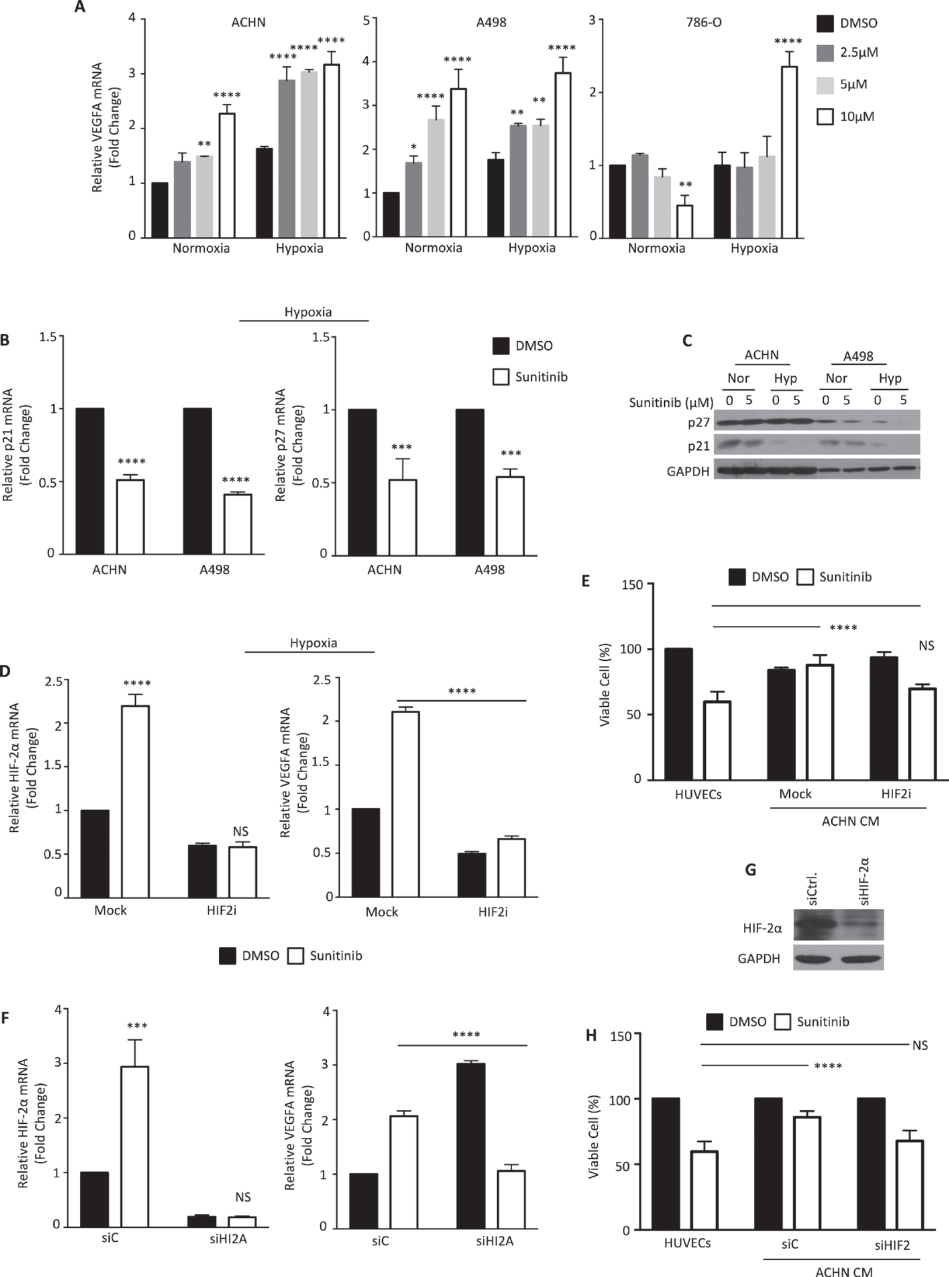
Sunitinib activation modulates HIF-2α levels (**A**) ACHN, A498 and 786-O cells were treated with the indicated dosages of sunitinib under normoxia or hypoxia for 24 h. *VEGFA* mRNA expression was determined by quantitative real-time PCR. (**B**) *p21* and *p27* mRNA expressions were analyzed at the indicated conditions by quantitative real-time PCR. (**C**) Protein levels were analyzed by Western blotting. (**D**) ACHN cells were incubated with DMSO (closed column) or 5 μM sunitinib (open column) and treated with or without an HIF-2α translation inhibitor (Merck 400087) under hypoxic condition. *HIF-2α* and *VEGFA* mRNAs were determined by quantitative real-time PCR. (**E**) HUVECs were cultured with condition medium derived from ACHN cells treated with HIF-2α translation inhibitor and incubated with DMSO (closed column) or 5 μM sunitinib (open column) for 24 h. (**F**) ACHN cells were treated with DMSO (closed column) or 5 μM sunitinib (open column) for 24 h, and mRNA levels were determined by quantitative real-time PCR. (**G**) ACHN cells were transfected with *HIF-2α* or negative control siRNA. The silencing effect on HIF-2α proteins was verified by Western blotting. (**H**) HUVECs were cultured with condition medium derived from ACHN cells transfected with *HIF-2α* or negative control siRNA and treated with DMSO (closed column) or 5 μM sunitinib (open column) for 24 h. The viability of HUVECs was measured by SRB assay. The results represent the means ± S.D. (*P ≤ 0.05, **P ≤ 0.01, ***P ≤ 0.001, ****P ≤ 0.0001, NS: *P* > 0.05).

In order to determine if HIF-2α is still functional in RCCs that have been treated with sunitinib, we assayed the expression of several known HIF-2α-regulated genes. We measured the mRNA levels of *VEGFA*, a common downstream target of both HIF-1α and HIF-2α, and *p21* and *p27*, two target genes activated by HIF-1α but inhibited by HIF-2α [23, 28]. In most growth conditions, sunitinib treatment increased *VEGFA* mRNA expression in RCCs in a dosage-dependent manner. This increase was especially prominent in RCCs grown under hypoxic conditions. However, sunitinib actually reduced *VEGFA* mRNA levels in a dosage-dependent manner in 789-O cells under normoxic conditions (Figure 3A). *p21* and *p27* expression levels were reduced in RCCs treated with sunitinib, especially in hypoxic A498 cells (Figure 3B and 3C). These results suggested that sunitinib treatment led to the down-regulation of HIF-1α and the up-regulation of HIF-2α function, which activated HIF-2α–dependent targets in RCCs.

Since sunitinib treatment enhanced HIF-2α transactivation activity in RCCs, we further tested whether HIF-2α was responsible for the protective effect against sunitinib provided by RCCs to HUVECs. We treated ACHN cells with an HIF-2α translational inhibitor for 48 h, which resulted in a decrease of *HIF-2α* mRNA levels (Figure 3D). *VEGFA* expression did not increase in ACHN cells treated with sunitinib and the HIF-2α translational inhibitor (Figure 3D). The cell growth rate was significantly suppressed by the HIF-2α translational inhibitor (Figure 3E).

To further confirm whether HIF-2α is responsible for the protective effect against sunitinib treatment provided to HUVEC cells by RCCs, we used small interfering RNA (siRNA) to knock down HIF-2α expression in ACHN cells. *HIF-2α* mRNA was efficiently depleted by siRNA compared to control siRNA, and the increase of *VEGFA* mRNA caused by sunitinib was completely blocked by *HIF-2α* knockdown (Figure 3F). Western blot analysis showed that the siRNA reduced the level of HIF-2α protein by around 60% (Figure 3G). We next obtained the conditioned medium from siHIF-2α and control siRNA ACHN cells treated with sunitinib for 24 h. The protective effect against sunitinib treatment provided to HUVECs by ACHN cells was completely compromised by the down-regulation of HIF-2α expression (Figure 3H). Our findings revealed that reciprocal regulation between HIF-1α and HIF-2α exists in RCC cells.

### Sunitinib promotes HAF-mediated HIF-1α ubiquitination and degradation

**Figure 4:**
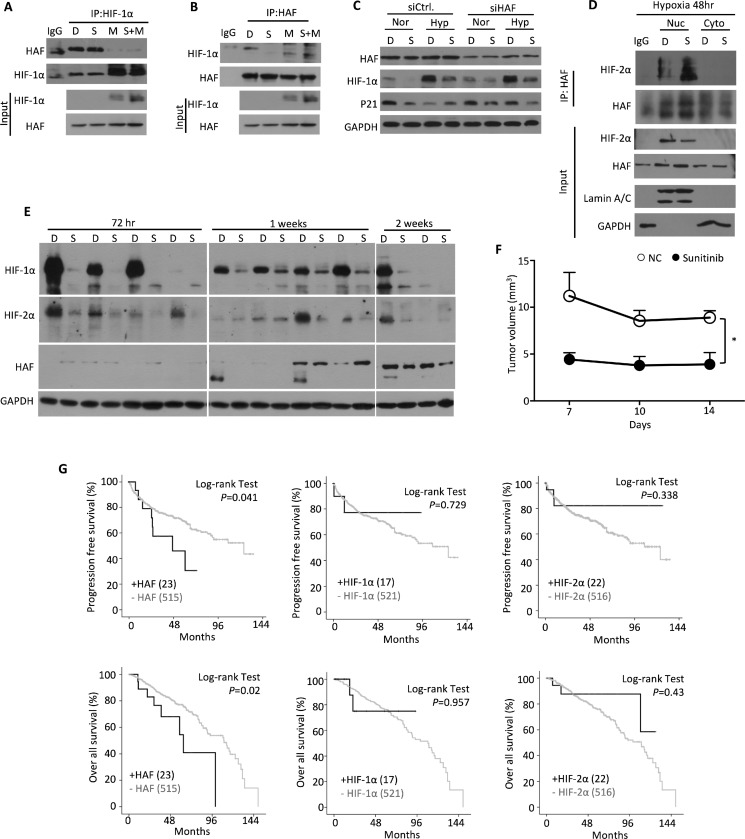
Characterization of the HAF-HIF-1α interaction under sunitinib treatment ACHN cells were treated with DMSO (D), 5 μM sunitinib (S) or/and 5 μM MG-132 (M) for 24 hours. Endogenous HIF-1α or HAF were immuno-precipitated by (**A**) HIF-1α antibody or (**B**) HAF antibody, and lysates were immuno-blotted with the indicated antibodies. (**C**) ACHN cells were transfected with *HAF* or negative control siRNA and treated with DMSO (D) or 5 μM sunitinib (S) in normoxia (Nor) or hypoxia (Hyp) for 24 hours. HAF, HIF-1α and p21 protein levels were analyzed at the indicated condition by Western blotting. (**D**) ACHN cells were treated with DMSO (D) or 5 μM sunitinib (S) and fractionated into nuclear (Nuc) and cytoplasmic (Cyto) parts. Fractionated lysates were immuno-precipitated by HIF-2α antibody and HAF antibody for Western blotting analysis with the indicated antibodies. (**E**) Mouse tumor masses were lysed with RIPA buffer. Total lysates were immune-blotted with the indicated antibodies. (**F**) Mouse tumor volumes were measured every three days. The results represent the means ± SEM. (*P ≤ 0.05). (**G**) Progression-free survival or overall survival rate of RCC patients was processed by Log-rank (Mantel-Cox) test. Human clinical data were from The Cancer Genome Atlas.

We observed that sunitinib treatment reduced the level of HIF-1α protein, but did not reduce the level of HIF-2α protein. We suspected the involvement of the E3 ubiquitin ligase HAF, because it has been shown to specifically ubiquitinate HIF-1α, but not HIF-2α [20, 22]. We determined whether sunitinib had an effect on the physical interaction between HIF-1α and HAF proteins, by using an immuno-precipitation assay in ACHN cells. Under sunitinib treatment, we observed that the interaction between HAF and HIF-1α increased, and that the degradation of HIF-1α protein was enhanced (Figure 4A and 4B). Similar amounts of HAF proteins were pulled down by HIF-1α antibody from sunitinib-treated cells as compared to the DMSO-treated control cells (Figure 4A). MG-132 interrupted most of interaction between HAF and HIF-1α proteins (Figure 4A). We observed lower amounts of HIF-1α precipitated by HAF antibody in cells treated with sunitinib alone than with sunitinib and MG-132 combined (Figure 4B), This suggested that sunitinib enhanced the physical interaction between the HAF and HIF-1α proteins, leading to more degradation of HIF-1α protein.

We further knocked down HAF to examine its functional role in HIF-1α protein degradation. Our results demonstrated that *siHAF* rescued the sunitinib-induced degradation of HIF-1α and p21 proteins under normoxia, but not under hypoxia (Figure 4C). We also observed that sunitinib increased the nuclear HIF-2α proteins in hypoxic ACHN cells (Figure 4D). To analyze the changes of HIF-1α and HIF-2α proteins *in vivo*, we treated mice with sunitinib through oral gavage. We observed a decreasing trend of HIF-1α protein levels and an increasing trend of HIF-2α and HAF protein levels over the duration of sunitinib treatment (Figure 4E). Additionally, we observed significant shrinkage of tumor mass over the course of treatment (Figure 4F). We further analyzed the clinical data to predict the roles of HIF-1α, HIF-2α, and HAF in tumor progression and survival rate (Figure 4G). Our findings suggested that only the expression status of HAF was involved in tumor progression and the patient survival rate.

### Identification of sunitinib-induced HIF-2α response cytokines

**Figure 5:**
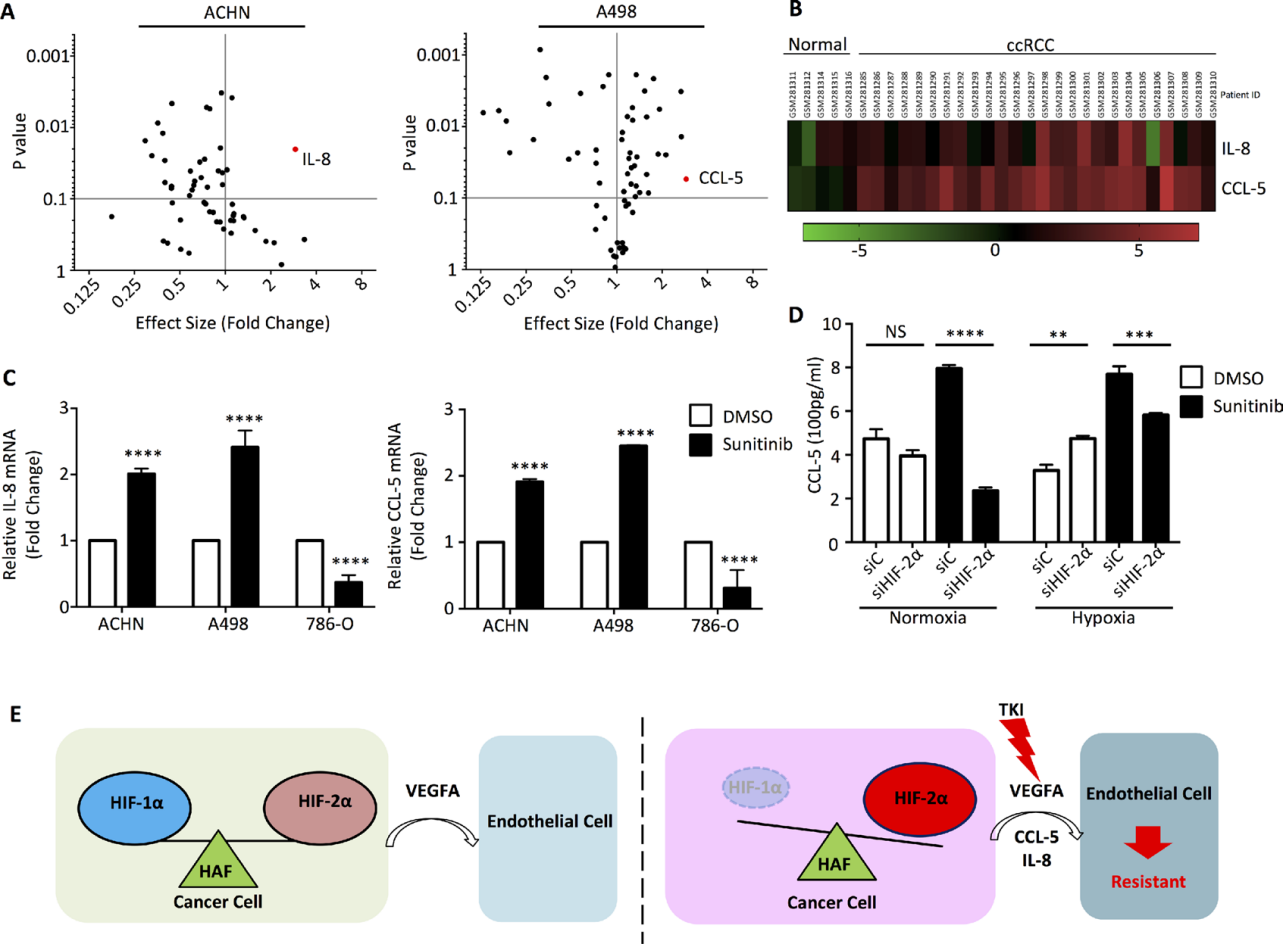
Cytokine RT-PCR Array identified specific cytokines involved sunitinib treatment (**A**) ACHN and A498 cells were incubated with DMSO or 5 μM sunitinib and total mRNA was extracted to perform RT-PCR primer array. Volcano plots illustrate the effect size by normalization with the DMSO negative control. (**B**) The heatmap of human clinical microarray profiling via Oncomine^™^ Platform Software shows *IL-8* and *CCL-5* mRNA expression levels. (**C**) The total mRNAs of ACHN, A498, and 786-O cells were analyzed for *IL-8* and *CCL-5* mRNA by quantitative real-time PCR. (**D**) A498 cells were transfected with *siHIF-2α* or negative control siRNA and treated with DMSO or 5 μM sunitinib in normoxia or hypoxia for 24 h. CCL-5 levels were analyzed by ELISA. (**E**) Schematic representation of how the microenvironment might provide RCCs escape from the inhibition of TKI to develop resistance effect.

To identify cytokines that have altered expression under sunitinib treatment, we performed RT-PCR on 88 cytokine/cytokine receptor related genes, using RNA from RCC cells treated with or without sunitinib. Among the 88 genes, *IL-8* and *CCL-5* were significantly induced by sunitinib (Figure 5A). From human cancer microarray data obtained from the Oncomine^TM^ Platform database, *IL-8* and *CCL-5* were shown to be up-regulated in ccRCC patients (Figure 5B). We then re-examined the functional role of HIF-2α on the expression of *IL-8* and *CCL-5* mRNAs in ACHN, A498, and 786-O cells treated with sunitinib. Under hypoxic conditions, *IL-8* and *CCL-5* mRNA levels increased under sunitinib treatment in ACHN and A498 cells, but decreased in 789-O cells (Figure 5C). Secreted CCL-5 in the culture medium of A498 cells was higher in cells treated with sunitinib, as measured by ELISA (Figure 5D). The increase in CCL-5 was mostly ameliorated when HIF-2α function was diminished by *siHIF-2α*, especially under normoxic conditions (Figure 5D).

## DISCUSSION

Hypoxia, a characteristic feature of solid tumors, has emerged as a pivotal factor in tumorigenesis. Hypoxia can activate genes involved in the adaptation of the tumor to its microenvironment, which promotes tumor progression and resistance to therapy [29, 30]. There are two general modes of resistance to anti-angiogenic TKIs therapy: adaptive (evasive) resistance and intrinsic (pre-existing) non-responsiveness [5, 6]. In this work, we sought out the mechanism(s) of evasive resistance to the current clinical therapy. Recently, there are at least four distinct adaptive mechanisms for evasive resistance to anti-angiogenic TKI therapy [6, 7]. As anti-angiogenic TKI therapy has been widely applied as a treatment for many kinds of advanced cancers, evasive resistance has emerged as a critical issue in clinical practice. Understanding the underlying mechanisms involved in the evasive resistance to anti-angiogenic TKI therapy is a prerequisite for long-term successful treatment outcomes of TKI. Here we provided several lines of evidence showing that HAF differentially degraded the HIF-1α protein and associated with HIF-2α protein, which drove evasive resistance to TKI therapy in most RCCs, except for the 786-O cell line. Our working model showed that RCCs modulate endothelial cells to be involved in angiogenesis in hypoxic conditions (Figure 5E). When cells were treated with TKIs, such as sunitinib in this work, the balance of HIF-1α and HIF-2α proteins were disrupted via the selective mRNA downregulation and protein degradation toward HIF-1α by HAF. Subsequently, HAF had the ability to functionally interact with HIF-2α and enhance its downstream targets activation, resulting in increased IL-8 and CCL-5 secretion by RCCs to stimulate endothelial cells growth and mediate the resistance to anti-angiogenesis TKI therapy. Lately, structure-based approach revealed that selective HIF-2α antagonist suppressed tumorigenesis in RCCs with multiple TKI resistance [31]. Hence, this study supporting our findings indicates that the targeting of the HAF–HIF-2α axis offers a promising therapeutic strategy for the future treatment of renal cell carcinoma.

Recently, chronic hypoxia has been implicated as a causal factor for the increased aggressiveness of tumors that develop resistance to anti-angiogenic therapy, such as VEGF inhibition [23, 32]. The link between anti-angiogenic TKIs therapy-induced hypoxia and bone marrow-derived cell recruitment might ablate blood vessels within a tumor and thereby cause acute hypoxia and necrosis [33, 34]. This triggers a transient accumulation of sufficient endothelial progenitor cells at the tumor margins to facilitate revascularization [35]. Our current data demonstrated that hypoxic status might also be an important factor in the development of sunitinib resistance; for example, the inhibition of p21 and p27 (Figure 3B and 3C). Therefore, rather than acting as a simple on-off switch as once thought, hypoxia initiates a complex cellular response that involves multiple players, including the HIFs, and depends on the duration and intensity of the hypoxia. Here, our findings also highlight the importance of the ratio of HIF-1α and HIF-2α in cells which up-regulates differentially transcribed target genes, such as *IL-8* and *CCL-5*, for specific physical (or pathological) functions.

Most of the target genes of the HIF family can be regulated by an oxygen- or pVHL-dependent mechanism in hypoxia [9]. Recently, HAF was reported to regulate the HIFs both in an oxygen- and pVHL-independent way [21, 23]. Hypoxia-dependent SUMOylation of HAF enables its binding to HIF-2α to promote the transcription of HIF-2α target genes without affecting HAFs ability to bind and degrade HIF-1α [21]. HAF switches cells from HIF-1α to HIF-2α via this subset of HIF-2α-dependent genes that drive tumor progression, and result in poor patient prognosis [21–24]. Sunitinib disrupted the balance between HIF-1α and HIF-2α due to the depletion of HIF-1α through mRNA suppression and protein degradation by the E3 ubiquitin ligase HAF. These unexpected findings can only be observed in RCCs expressing both HIF-1α and HIF-2α, and illustrate out how RCCs protect HUVECs against sunitinib treatment, which may contribute to the sunitinib resistance phenotype. HAF might not only be involved in the disruption of the balance between HIF-1α and HIF-2α in the sunitinib treatment, but also in the regulation of HIF-2α-dependent transactivation for the growth protective effect of RCCs upon sunitinib treatment. Further experiments are needed to determine whether sunitinib modulates the SUMOylation status of HAF during the functional interactions between HIF-2α and HAF.

Here, our work demonstrates the novel possibility of evasive resistance to TKI therapy in the current clinical RCCs therapy. The subtle change in the ratio of HIF-1α and HIF-2α in cells, via the dual functions of HAF in hypoxia, might provide a new strategy to develop a combination therapy for renal cell carcinoma. Therefore, finding a specific HIF-2α inhibitor should be a future research aim.

## MATERIALS AND METHODS

### Cell culture

The ACHN, A498 and 786-O cells were obtained from the Bioresource Collection and Research Centre, Taiwan. The cells were cultured in culture media as recommended by the American Type Culture Collection and at 37°C in a 5% CO_2_, 95% humidity incubator.

### SRB assay

The cells were plated in 96-well plates at a plating density of 3000 cells/well. After 24–72 hours with TKIs treatment, the cells were fixed with 10% trichloroacetic acid (Sigma-Aldrich, St. Louis, MO, USA) and stained with 0.4% SRB (Sigma-Aldrich, St. Louis, MO, USA). The plates were read in a Perkin-Elmer Victor micro-plate reader at 570 nm, as described previously [36]. Triplicate wells per condition were evaluated and the data presented were representative of three independent experiments.

### Conditioned medium and co-culture assay

TransWell^®^ chambers with 0.4 μm pores were loaded with 1 × 10^5^ of HUVECs, ACHN, A498 or 786-O cells in the inserts and 1 × 10^5^ HUVECs in the wells. After 24 hours, TKIs (sorafenib, sunitinib, axitinib, or pazopanib) were added to the wells and the culture system kept for 24 hours. Afterward, HUVECs were analyzed for cell viability via SRB assay. For the conditioned medium assay, 1 × 10^6^ of HUVECs, ACHN, A498, or 786-O cells in the 10 cm^2^ dish were cultured with sorafenib (5, 10 and 15 μM), sunitinib (0.1, 0.5 and 1 μM), axitinib (5, 10 and 20 nM) and pazopanib (5, 10 and 20 nM) under normoxia or hypoxia (1% O_2_) for 24 hours. Afterward, the culture medium as well as the conditioned medium was collected and centrifuged at 10,000 rpm, 4°C 10 mins to remove debris. HUVECs (1 × 10^3^) were incubated in the conditioned medium for 24 hours and then cell viability was analyzed via SRB assay.

### SiRNA and transfection

*HIF-2α* siRNA, *HAF* siRNA, or negative control siRNA (ON-TARGET plus non-targeting pool, Dharmacon) were transfected into cells with 100 nM Lipofectamine^TM^ 2000 (Invitrogen Life Technologies, Carlsbad, CA). Culture media containing the reagent mixture were removed and replaced with fresh complete medium after 8 h transfection, incubated for 16–18 h, and then used for further experiments.

### Quantitative real-time PCR

Complementary DNA (cDNA) was synthesized using Superscript II^®^ reverse transcriptase (Invitrogen Life Technologies, Carlsbad, CA) following the manufacturer's recommendations, except that the addition of RNase inhibitor was omitted. cDNA was diluted 1:10 in sterile water. Quantitative PCR was performed on a LightCycler**^®^** 480 System (Roche) using SYBR Green PCR reagents in a 50 ml reaction mixture containing 5 ml 10× SYBR Green PCR Buffer, 0.5 μl 10 mM primers, 4 μl dNTP mix, 6 μl 25 mM magnesium chloride, 0.5 μl AmpErase, 0.25 μl Amplitaq Gold and 5 μl of the 1:10 diluted cDNA synthesis reaction product. PCR was performed for 40 cycles at 95°C for 15 seconds and 60°C for one minute after initial incubations at 50°C for 2 minutes and 95°C for 10 minutes. PCR product specificity and purity were evaluated by generating a dissociation curve following the manufacturer's recommendations. Sample Ct values were normalized to Ct values for GAPDH RNA, all of which were calculated from triplicate reactions. The following primer pairs were used: HIF-1α,5′-ATCCATGTGACCATGAGGAAATG-3′ and 5′-TCGG CTAGTTAGGGTACACTTC-3′; HIF-2α,5′-GGACTTA CACAGGTGGAGCTA-3′ and 5′-TCTCACGAATCT CCTCATGGT-3′; VEGFA, 5′-TTATGCGGATCAAAC CTCACC-3′ and 5′-GAAGCTCATCTCTCCTATGTG C-3′; IL-8, 5′-ATGACTTCCAAGCTGGCCGTGGCT-3′ and 5′-TCTCAGCCCTCTTCAAAAACTTCTC-3′; CCL-5, 5′-TGCATCTGCCTCCCCATATTC-3′ and 5′-CTTCTC TGGGTTGGCACACA-3′; p21, 5′-TGTCCGTCAGAA CCCATGC-3′ and 5′-AAAGTCGAAGTTCCATCGCT C-3′; p27, 5′-AACGTGCGAGTGTCTAACGG-3′ and 5′-CCCTCTAGGGGTTTGTGATTCT-3′; HAF, 5′-CCA GCTCCAAAACTAGCTCAG-3′ and 5′-AAGGCCATA GGGTTGATGACA-3′. Relative gene induction values were calculated following the manufacturer's recommendations.

### Western blotting

Whole cell extracts were analyzed by SDS-PAGE and transferred to a nitrocellulose membrane using a transfer apparatus according to the manufacturer's protocols (Bio-Rad). After incubation with 10% non-fat milk in TBST (10 mM Tris, pH 8.0, 150 mM NaCl, 0.1% Tween 20) for 1 hour, the membrane was washed once with TBST and incubated with antibodies against HAF (1:2000, Santa Cruz), HIF-1α (1:2000, BD Biosciences), HIF-2α (1:1000, Novus Biologicals), p27 (1:1000, Santa Cruz), p21 (1:1000, Santa Cruz) or GAPDH (1:1000, Sigma-Aldrich) at 4°C for 12 h. Membranes were washed three times for 10 min and incubated with a 1:5000 dilution of horseradish peroxidase-conjugated anti-mouse or anti-rabbit antibodies for 2 h. Blots were washed with TBST for 1 hour and developed with the ECL system (Millipore) according to the manufacturer's protocols.

### Cytokine RT-PCR primer array

Cells were treated with sunitinib or DMSO for 24 hours, then mRNA was collected and used to synthesize cDNA for Cytokine-cytokine receptor interaction primer array (TAKARA). Synthesized cDNA and SYBR® Premix Ex Taq ™ II (Perfect Real Time) were combined to prepare a master mix solution for control and test samples, and then dispensed into the wells of a 96-well real-time PCR plate. Afterward, primers of the PrimerArray^™^ were added to the real-time PCR plate using 8-multichannel pipette. The real-time PCR plate was placed in a real-time PCR instrument, and the following program was started following the initial denaturation: 95°C for 30 sec and elongation: 95°C for 5 sec and 60°C for 30 sec (40 cycles).

### Subcellular fraction

Cells were separated into cytosolic and nuclear fractions by modifying the protocol of a subcellular protein fractionation kit (Pierce Biotechnology, USA). After 24 hours of drug incubation, 15 cm^2^ of cells were pelleted by centrifuging at 700× g for 5 min. Cells were washed using ice-cold PBS and centrifuged at 700 x g for 5 min. Cells were suspended with ice-cold cytosolic lysis buffer. Supernatants were collected for cytosolic extracts. Pellets were re-suspended by using nuclear lysis buffer. After centrifugation at 12000× g for 15 min, supernatants were transferred to chilled 1.5 ml tubes as nuclear extracts.

### ELISA

The cells were transfected with siRNA or negative control and incubated with or without sunitinib. The measurement of CCL-5 was performed as described by the Human SimpleStep ELISA Kit (Abcam, USA).

### *In vivo* xenograft model

Care of the animals was in accord with our institutional guidelines. Cancer cells (1 × 10^7^) were injected subcutaneously on the right flank of 4–6 week old female athymic nude mice (National Laboratory Animal Center, Taiwan). Athymic mice are immune-deficient and cannot develop a complete adaptive immune response, but have complement and NK cell activities. The drug was delivered orally at 60mg/kg/day. Tumor growth was measured every 3 days. Tumor volume was calculated using the formula: Volume = length × width^2^ × 0.5. Tumors were allowed to reach about 200–250 mm^3^ before randomization.

### Statistical analysis

Data were analyzed by Student's *t-test*. A *p value* of less than 0.05 was considered as statistically significant.
